# Five dichotomies in the psychophysics of ensemble perception

**DOI:** 10.3758/s13414-020-02027-w

**Published:** 2020-04-29

**Authors:** Joshua A. Solomon

**Affiliations:** Centre for Applied Vision Research, City University of London, London, EC1V 0HB UK

**Keywords:** signal detection theory, Spatial Vision, statistics

## Abstract

Whereas psychophysicists may formulate hypotheses about appearance, they can only measure performance. Bias and imprecision in psychophysical data need not necessarily reflect bias and imprecision in perception. Sensory systems may exaggerate the differences between each item and its neighbors in an ensemble. Alternatively, sensory systems may homogenize the ensemble, thereby removing any apparent differences between neighboring items. Ensemble perception may be involuntary when observers attempt to report the identities of individual items. Conversely, when asked to make a (voluntary) decision about the ensemble as a whole, observers may find it very difficult to compute statistics that are based on more than a very small number of individual items. Modeling decisions about prothetic continua, such as size and contrast, can be tricky because sensory signals may be distorted before and/or after voluntarily computing ensemble statistics. With metathetic continua, such as spatial orientation, distortion is less problematic; physically vertical things necessarily appear close to vertical and physically horizontal things necessarily appear close to horizontal. Decision processes are corrupted by noise that, like distortion, may be added to sensory signals prior to and/or after voluntarily computing ensemble statistics.

The ginkgo leaves fell like fine rain from the boughs and dotted the lawn with yellow. . . . I said I would like to distinguish the sensation of each single ginkgo leaf from the sensation of all the others, but I was wondering if it would be possible.

–Italo Calvino, *If on a winter’s night a traveler* (1998/1979, p. 199)

There are two types of people: Those who see dichotomies everywhere, and I’m one of them. It is my hope that some of what follows may serve as a tutorial to students of ensemble perception. Readers of this listicle should be forewarned: Most of the examples provided below were produced in my own laboratory. Whitney and Yamanashi-Leib (2018) offer a more balanced review.

## Appearance v performance

The epigraph suggests that individual sensations may depend on whether they form part of an ensemble. The author of this passage is a character in Calvino’s book. His suggestion is almost certainly correct, but I want to underscore the fact that psychophysicists are fundamentally incapable of directly measuring sensation. Physiologists can directly measure neural output, but all a psychophysicist can do is measure visual performance. The ensemble may bias our perception of any individual ginkgo leaf, but bias and imprecision in psychophysical data need not necessarily reflect bias and imprecision in perception.

As an example, consider Brady and Alvarez’s (2011) investigation of the effect of ensemble membership on reproduction error. On each trial of their experiment, human observers resized a circle (with a computer mouse) to match one member of a previously seen ensemble of circles. Errors tended to be in the direction of the ensemble mean, but Brady and Alvarez did not unnecessarily infer any homogenization of perceived sizes within the ensemble. Although each individual circle’s size may have appeared closer to that of the ensemble mean, Brady and Alvarez concluded that reproductions were consistent with a type of rational decision-making, under uncertainty, given the limited capacity of visual working memory.

One of the most pernicious pitfalls in attempting to infer appearance from behaviour is the subject-expectancy effect (e.g., Rosenthal & Rubin, 1978), in which experimenter expectations are transmitted to the participants in such a way as to alter the outcome of the experiment in a desired direction. There is, perhaps, no way to preclude nonperceptual biases from contaminating psychophysical estimates of appearance, but certain measures can be taken to minimize their likelihood and/or severity.

One strategy is to use a psychophysical paradigm in which observers’ response categories are decoupled from experimenter expectations. For example, Morgan, Melmoth, and Solomon (2013) expected that vertically aligned spots would appear to have a clockwise tilt, when they were displayed within a square frame having a counterclockwise tilt. Rather than asking observers to report the apparent tilt of the spots, they asked observers to inspect two sets of spots and report which set was closest to vertical. Sometimes both sets were clockwise, sometimes both sets were counterclockwise, and sometimes one set was clockwise of vertical while the other set was counterclockwise. Variants of this “comparison-of-comparisons” strategy have become increasingly popular (e.g., Ismail, Solomon, Hansard,, & Mareschal, 2016; Jogan & Stocker, 2014; Maloney & Yang, 2003; Morgan, Grant, Melmoth, & Solomon, 2015; Morgan, Schreiber, & Solomon, 2016; Yarrow, Martin, Di Costa, Solomon, & Arnold, 2016).

Also popular are “double-blind” strategies, where steps are taken to remove the bias-inducing stimuli from the observer’s awareness. For example, it is possible to observe a tilt aftereffect after adapting to gratings with unresolvably high spatial frequency (He & MacLeod, 2001). The tilt aftereffect also survives removal of the adapting stimulus from awareness by crowding (defined below; He, Cavanagh, & Intrilligator, 1996) and critical-flash suppression (Bahrami, Carmel, Walsh, Rees, & Lavie, 2008).

One final strategy is to make performance depend upon appearance. Inferences regarding the latter can be made from objective measurements of the former. For example, Morgan and Solomon (2019) asked observers to select the odd man out in a variety of visual search tasks, where performance is determined by the target’s relative salience (i.e., how different it appears from the distractors). Adaptation was used to manipulate target salience, and aftereffect strength was inferred from the degree to which performance was facilitated or impaired by adaptation.

## Repulsion v assimilation

Although it may be relatively trendy to consider how ensemble membership affects the appearance of its individual members, studies relating an item’s appearance to its general spatial and temporal context are nothing new. The motion aftereffect, for example, was described by Addams (1834), who reported that the nearby rocks appeared to move upwards, after adapting to the downward motion of the Falls of Foyers. Chevreul (1855) described the exaggeration of simultaneous luminance contrast between neighbouring stripes with different grey levels (p. 8). The general term for these sensory phenomena is “repulsion,” and it occurs in every feature domain I have examined.

**Fig. 1.**
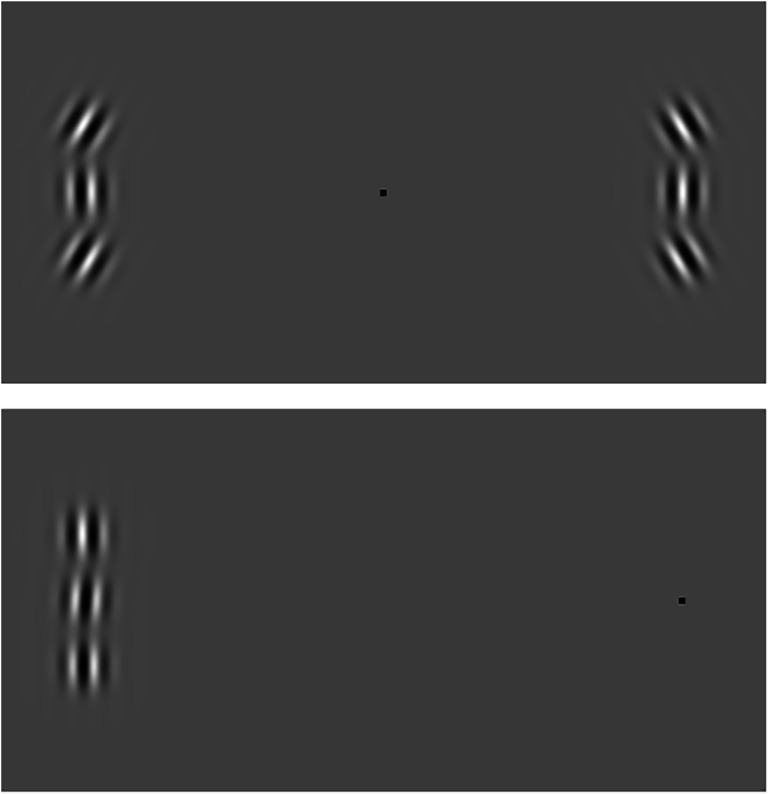
Demonstrating repulsion and assimilation. The visual system exaggerates the orientation contrast between the (45°) tilted flankers and the vertical elements in the top panel. Small orientation differences, on the other hand, are effectively squelched. Thus, vertical flankers in the bottom panel can cause the small (4°) tilt to go unnoticed

Figure 1 (top panel) shows an example of the visual system exaggerating the simultaneous orientation contrast between neighbouring Gabor patterns. The truly vertical central elements on either side of the black fixation spot should appear to be tilted in opposite directions when you stare directly between them. This can be described as an exaggeration of the difference between the orientation of each central element and the orientation of its flanking distractors. This exaggeration is usually pretty large for briefly presented stimuli (i.e., short flashes; Wenderoth & van der Zwan, 1989), but it also depends on the retinal eccentricity of the central Gabor patterns.

**Fig. 2.**
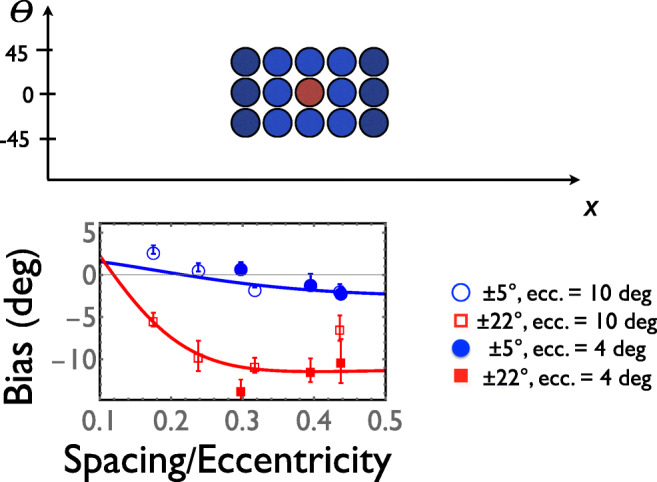
Repulsion and assimilation: theory and experiment. Top panel contains a cartoon of 15 “classical” receptive fields somewhere in visual cortex. Axes are one dimension of visual space (e.g., horizontal position *x*) and orientation preference *θ*. Horizontal stimuli positioned so as to maximally stimulate the central (red) cell indirectly and strongly inhibit nearby (light blue) cells and weakly inhibit the somewhat further (dark blue) cells, via lateral connections. Reciprocality in those connections means that the latter cells can also inhibit the central one. Thus, the amalgamation of these 15 receptive fields can be considered the “extra-classical” receptive field of the central cell. The lower panel shows average biases (symbols; ±1 standard error) and model fits (solid and dashed curves) to Experiment 3 from Mareschal, Morgan, and Solomon (2010), in which observers judged the orientation (with respect to a horizontal reference) of a flanked Gabor pattern at 4 and 10 degrees of retinal eccentricity. All flankers were identically rotated either −22°, −5°, +5°, or +22° with respect to the horizontal. (Colour figure online)

Exaggerations of simultaneous contrast are routinely ascribed to lateral inhibition somewhere within the visual system. The cartoon at the top of Fig. 2 represents 15 receptive fields somewhere in visual cortex, with lateral inhibition spreading around the cell whose receptive field is red. If lateral inhibition is responsible for the exaggeration of simultaneous contrast, then we have psychophysical data suggesting that lateral inhibition spreads very far indeed. Consider the red symbols in the lower half of Fig. 2. They represent trials in which flankers were tilted either 22° degrees clockwise or 22° degrees counterclockwise of horizontal. When these flankers were present, they produced a negative or “repulsive” bias of roughly 10° when observers attempted to report the orientations of near-horizontal targets. This was true even when the distance between flanker and target was almost half the latter’s eccentricity.

The blue symbols, on the other hand, represent trials in which the flankers were tilted just 5° off horizontal. When those flankers were close to the target, they seemed to produce a positive bias. This assimilation means that the visual system effectively makes it hard to notice subtle changes in orientation (and any other feature dimension you care to name), particularly when you do not look directly at the stimulus. Orientation assimilation is demonstrated in Fig. 1b. You might need to look slightly to the left or right of the black spot, but you should be able to find a fixation for which you can still see all three Gabors without being able to see the differences in their orientations.

## Involuntary averaging v voluntary averaging

Whatever is responsible for small-angle assimilation, it too extends over a vast swathe of visual cortex. The only reason small-angle assimilation disappears when flankers and target are widely separated is because lateral inhibition is stronger. To get around the problem of lateral inhibition, some researchers have adopted tasks that should be immune to it. That is, they use visual tasks that do not require making fine discriminations between similar orientations or luminances or whatever. One task they particularly like is letter identification.

**Fig. 3.**
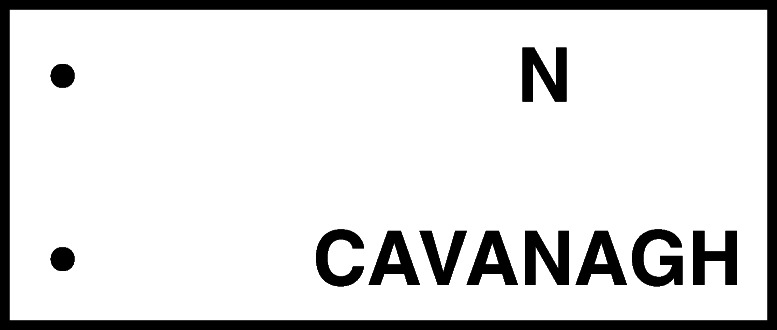
Crowding. When fixating the dot on the top left, the N to the right is easily identified. When fixating the dot just below, the N seems to meld into a jumbled texture. Adapted from Cavanagh (2001)

The idea behind Fig. 3 is that the visual system is compelled to combine information from discrete stimuli, and consequently cannot disentangle the identity of one individual element from the overall statistics of information coming from that region of the visual field. In general, the name given to this phenomenon is “crowding,” and experiments with letter identification suggest a compulsory combination of visual information from individual letters separated by anything up to half their average eccentricity (Bouma, 1970).

Parkes, Lund, Angelucci, Solomon, and Morgan (2001) described crowding as “texture perception when we do not wish it to occur.” (p. 742). In other words, crowding is a form of involuntary ensemble representation. It is evident only when the visual task requires a decision about individual visual objects. I would like to distinguish such tasks from those that require a decision about the ensemble as a whole. In such cases, the computation of ensemble statistics may very well be voluntary.

When investigating ensemble representations, most of my work has been on voluntary averaging. For example, Solomon and Morgan (2018) recently assessed how well observers could estimate the average number of dots in a dot patch. The metric we used was efficiency. The paradigm was two-(temporal)-alternative forced choice: observers were asked whether the dot patches in the first ensemble had greater or fewer dots on average than patches in a second ensemble. Obviously, if both ensembles had the same number of patches, then the question would be identical to asking which stimulus had more dots. However, in some blocks of trials, the first ensemble had eight patches, while the second had just four patches. We also had blocks of four followed by eight. A binomial distribution was sampled to determine the number of dots in each patch. To compute efficiency, you need to calculate the ideal performance with various fractions of the available information. For example, if human performance were similar to what an ideal observer could do with half the available information, then human efficiency would be 50%.

**Fig. 4.**
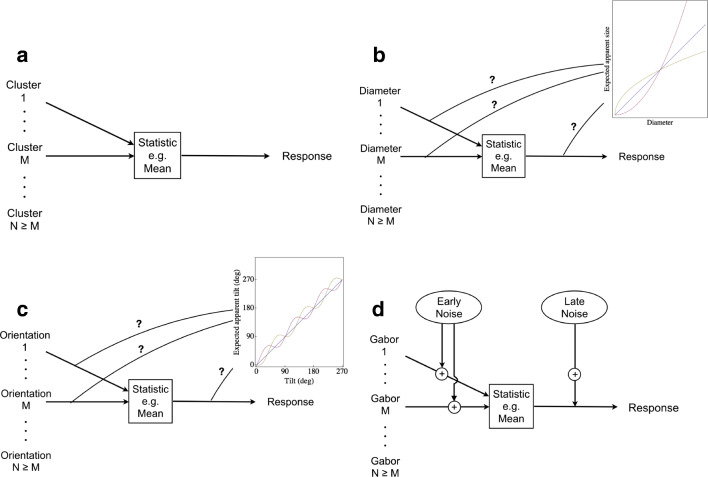
The inefficient observer model applied to voluntary averaging of (**a**) numerosities, (**b**) sizes, and (**c**) orientations. Psychophysical transformations (from physical dimensions into visual signals) are shown in the insets of Panels **b** and **c**. Note these transformations (expansive, compressive, or quasilinear) may occur prior to or after statistical summarization. Panel (**d**) shows the noisy, inefficient observer model with both (presummarization) early and (postsummarization) late sources of noise. (Colour figure online)

Figure 4a shows a flow diagram of the inefficient observer model for voluntary averaging. There were *N* dot clusters, observer J.A.S. selected *M* of these at random, measured their numerosities, and reported the mean of those *M* measurements. *M* is known as the effective sample size and the ratio of *M*:*N* is known as either the sampling efficiency or the calculation efficiency or simply “the efficiency.” For J.A.S., *M* was about 3.

## Prothetic v metathetic continua

The inefficient observer model can be traced back to one (Dakin, 2001) of the two papers that massively popularized the study of ensemble statistics. Dakin (2001) studied voluntary averaging of orientation, and Ariely (2001) studied voluntary averaging of size. Subsequently, I studied both, but orientation is easier, because it is a metathetic continuum.

The distinction was made by Stevens (1957). Simply put, prothetic continua are those such as size and contrast, where one value is either greater than, equal to, or less than another. Metathetic continua, such as orientation and direction, do not work like that.^1^ Prothetic continua such as size can be tricky to work with, because apparent size may not scale linearly with physical size. Indeed, without sufficiently specific instructions (cf. Raidvee, Toom, Averin, & Allik, 2020) it is not necessarily clear whether average size reflects average diameter, average area, or something in between.

Moreover, the transformation between physical size and apparent size could theoretically occur before and/or after voluntarily computing whatever statistic we wanted to study. The inset in Fig. 4b shows three psychophysical (transducer) functions. The blue one is linear, the violet one is expansive, and the yellow one is compressive.

Solomon and Tyler (2018) studied voluntary averaging in the contrast domain. When asked to adjust the contrast of a static, black-and-white texture to match the average contrast of another black-and-white texture that was flickering between high and low contrast, all observers gave the static texture greater average contrast than the flickering texture. This result is consistent with an accelerating or expansive transformation of physical contrast, prior to voluntary averaging.

With metathetic continua, like orientation, transformations like this do not really matter, because we can be pretty confident that physically vertical things appear close to vertical and physically horizontal things appear close to horizontal. Thus, the psychophysical function for orientation may stray a little from the blue line of veridicality in Fig. 4c, but it cannot stray too far.

## Early v late noise

**Fig. 5.**
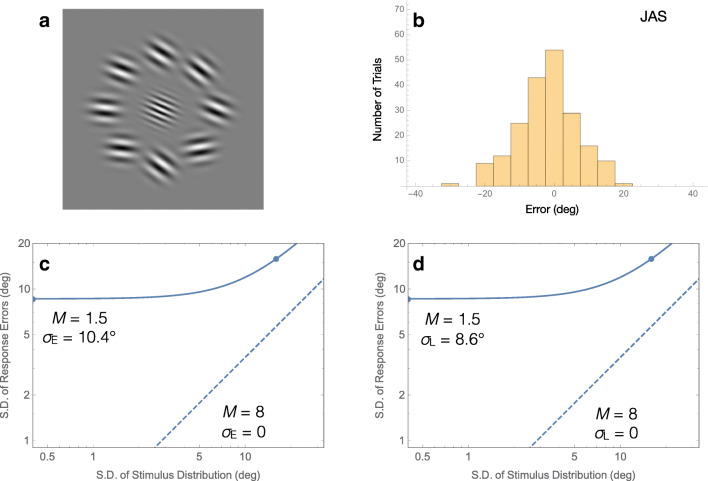
Voluntary averaging of orientation. **a** Stimulus from Solomon and Tyler (2015). Observers adjusted the central probe to match their memory of the surrounding ensemble’s average orientation. **b** Histogram of a representative observer’s adjustment errors when the standard deviation of low-frequency Gabor orientations was zero. **c–d** Threshold vs. noise (in this case, the standard deviation of adjustment errors v the standard deviations of the distribution of ensemble orientations) plots, fit with the noisy, inefficient observer model assuming zero late noise (i.e., *σ*_L_ = 0; **c**) and zero early noise (i.e., *σ*_E_ = 0; **d**). Smooth curves are maximum-likelihood fits to thresholds (dots) from J.A.S. Dashed lines illustrate the ideal observer

Perhaps the most direct way to test how well an observer can voluntarily compute an average is to ask the observer to reproduce that average. Solomon and Tyler (2015) showed observers an ensemble comprised of eight low-frequency Gabor patterns in a ring around fixation (see Fig. 5a). Their orientations were drawn from a wrapped normal distribution. The mean of that distribution was totally random, but its standard deviation was either zero or 16°. The ensemble was visible for 0.42 s. A high-frequency “probe” Gabor was presented at fixation after the ensemble had disappeared. Observers rotated the probe until its orientation matched their memory of the ensemble’s average orientation.

Although the inefficient observer model (see Fig. 4a) is useful in some cases, in general, it is not a good model of human performance, because humans cannot measure feature values like orientation with infinite precision. If they could, then response errors would have dropped to zero when all eight of the low-frequency Gabors were parallel. Errors do not drop to zero, as can be seen in Fig. 5b. Thus, the inefficient observer model needs modification. Perhaps the most straightforward way to modify the model is to assume that each measurement made by the observer gets perturbed by an independent, identically distributed sample of noise, as illustrated in Fig. 5c.

This noisy, inefficient observer model for voluntary averaging (and other summary statistics) has two free parameters: the effective set-size *M*, and the standard deviation of noise *σ*_E_:

1 $$ \mathrm{error}\kern0.5em \mathrm{S}.\mathrm{D}{.}^2=\frac{\sigma_{\mathrm{E}}^2+\mathrm{stimulus}\kern0.5em \mathrm{S}.\mathrm{D}{.}^2}{M}. $$

Within this simple formula, the standard deviation of response errors increases as a function of the standard deviation of orientations in the ensemble. A fit to one observer’s data is shown in Fig. 5c’s threshold-v-noise plot. The axes are logarithmic, so data from the condition with parallel Gabors appears at the leftmost edge of the plot. When the model is best fit to these two data points, its noise has a standard deviation of 10.4 degrees. The effective set size is 1.5.

Noninteger values such of effective set size can be interpreted in two ways. One possibility is that they reflect a mixture of effective set sizes. For example, on some trials, I might have ignored or forgotten all but one randomly selected element, whereas on other trials, I computed the average of two or more. In this case, *M* would reflect the root mean square of this number. Alternatively, noninteger values of *M* might reflect an unequal weighting of two or more of the texture elements.^2^

The smooth curve in Fig. 5d illustrates a slightly different noisy, inefficient observer:

2 $$ \mathrm{error}\kern0.5em \mathrm{S}.\mathrm{D}{.}^2={\sigma}_{\mathrm{L}}^2+\frac{\mathrm{stimulus}\kern0.5em \mathrm{S}.\mathrm{D}{.}^2}{M} $$

In this case, it is as though the entire ensemble gets tilted by a random amount, rather than each individual element. The standard deviation of that random amount *σ*_L_ is called “late noise” in this formulation.^3^ The first thing to notice is that the two versions of the model make exactly the same predictions. The second thing to notice is that the best fitting value of *M* does not depend on whether individual samples of equivalent noise are correlated. Thus, if all you care about is the effective set size, you can use either formula—it does not matter.

I attempted to ascertain whether the noise that limited the precision of orientation statistics was early or late (Solomon, 2010). This would have been impossible if I had merely asked observers to judge the average orientation of ensembles having a fixed size. Consequently, I varied the size of the ensemble, taking care not to change the precision with which the orientation of each individual element could be assessed, and I also varied the task: In some trials, observers were asked to assess the variance of orientations in the ensemble, rather than the mean. The results contained compelling evidence for both early and late noise. When members of an ensemble are all roughly identical, any effect of sample size *N* on the precision of voluntary averaging must be attributed to early noise. On the other hand, late noise is implicated when the effect of the sample’s size increases with the sample’s variance.

In conclusion, I would like to underscore two features/caveats with regard to the efficiency metric. First, it is a purely descriptive measure of performance in relation to the available information. Given any sample size *N*, efficiency is the ratio of *M* to *N*, where *M* is the sample size that the ideal observer would need in order to estimate a statistic with the same precision as a human observer. Since the ideal observer is, by definition, at least as good as any other observer, human decisions are necessarily based on *M* or more elements. Second, and most important, estimates of efficiency tell us nothing about ensemble perception per se. With strategic decision-making strategies, reasonably high efficiencies can be achieved from independent representations of individual stimuli. Authors discussing various strategies include Myczek and Simons (2008); Solomon (2010); Dayan and Solomon (2010); Gorea, Belkoura, and Solomon (2014); and Li, Herce Castañon, Solomon, Vandormael, and Summerfield (2017).

## Footnotes

1 “Continua having to do with *how much* belong to what we have called Class I, or prothetic; continua having to do with *what kind* and *where* (position) belong to Class II, or metathetic” (italics as in Stevens, 1957, p. 154)

2 The inefficient observer model can be considered an extreme form of unequal weighting, in which *N* − *M* elements get zero weight. Whereas any form of unequal weighting is subideal and thus inefficient, equal weighting does not imply 100% efficiency. An observer whose responses on each trial are based on a single, randomly selected element will have an efficiency of 1/*M*, but—on average—weight will have been distributed equally over all elements in the sample.

3 Note that segregating the cause of imprecision into an early and late stage is somewhat arbitrary. If instead of late noise we allow the random perturbation added to the orientation of any two elements to have correlation *ρ*, then we can reparameterize Equations 1 and 2 such that:

3 $$ \mathrm{error}\kern0.5em \mathrm{S}.\mathrm{D}{.}^2=\frac{\rho \left(M-1\right)}{M}{\sigma}^2+\frac{\sigma^2+\kern0.5em \mathrm{stimulus}\kern0.5em \mathrm{S}.\mathrm{D}{.}^2}{M} $$

Note that when *ρ* = 0, Equation 3 reduces to Equation 1 with *σ = σ*_E_; when ρ = 1, Equation 3 reduces to Equation 2 with *σ = σ*_L_.
